# Heavy‐Atom‐Free Room‐Temperature Phosphorescent Rylene Imide for High‐Performing Organic Photovoltaics

**DOI:** 10.1002/advs.202103975

**Published:** 2021-11-23

**Authors:** Ningning Liang, Guogang Liu, Deping Hu, Kai Wang, Yan Li, Tianrui Zhai, Xinping Zhang, Zhigang Shuai, He Yan, Jianhui Hou, Zhaohui Wang

**Affiliations:** ^1^ College of Physics and Optoelectronics Faculty of Science Beijing University of Technology Beijing 100124 P. R. China; ^2^ Key Laboratory of Organic Optoelectronics and Molecular Engineering Department of Chemistry Tsinghua University Beijing 100084 P. R. China; ^3^ Institute of Chemistry Chinese Academy of Sciences Beijing 100190 P. R. China; ^4^ Key Laboratory of Luminescence and Optical Information Ministry of Education School of Science Beijing Jiaotong University Beijing 100044 P. R. China; ^5^ Department of Chemistry and Energy Institute Hong Kong University of Science and Technology Clear Water Bay, Kowloon 999077 Hong Kong

**Keywords:** excited state characteristics, organic photovoltaics, room‐temperature phosphorescent electron acceptor, rylene imides

## Abstract

Organic phosphorescence, originating from triplet excitons, has potential for the development of new generation of organic optoelectronic materials. Herein, two heavy‐atom‐free room‐temperature phosphorescent (RTP) electron acceptors with inherent long lifetime triplet exctions are first reported. These two 3D‐fully conjugated rigid perylene imide (PDI) multimers, as the best nonfullerene wide‐bandgap electron acceptors, exhibit a significantly elevated T_1_ of ≈2.1 eV with a room‐temperature phosphorescent emission (*τ* = 66 µs) and a minimized singlet–triplet splitting as low as ≈0.13 eV. The huge spatial congestion between adjacent PDI skeleton endows them with significantly modified electronic characteristics of S_1_ and T_1_. This feature, plus with the fully‐conjugated rigid molecular configuration, balances the intersystem crossing rate and fluorescence/phosphorescence rates, and therefore, elevating *E*_T1_ to ≈2.1 from 1.2 eV for PDI monomer. Meanwhile, the highly delocalized feature enables the triplet charge‐transfer excitons at donor–acceptor interface effectively dissociate into free charges, endowing the RTP electron acceptor based organic solar cells (OSCs) with a high internal quantum efficiency of 84% and excellent charge collection capability of 94%. This study introduces an alternative strategy for designing PDI derivatives with high‐triplet state‐energy and provides revelatory insights into the fundamental electronic characteristics, photophysical mechanism, and photo‐to‐current generation pathway.

## Graphical abstract

The long‐lived excitons are of significance to alleviate the strong morphology‐ and thickness‐dependence of organic photovoltaic performance and therefore accelerating its industrialization process. Here, two room‐temperature phosphorescent electron acceptors with long‐lived triplet exctions and high‐energy level are investigated systematically for the first time. This work provides revelatory insights into their fundamental electronic characteristics, photophysical mechanism, and photo‐to‐current generation pathway.

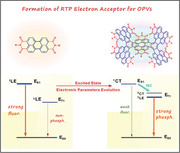

## 1 Introduction

The luminescence of organic materials is an important photophysical process that is fundamentally important for practical applications in the fields of sensing,^[^ ^1^ ^]^ color displays,^[^ ^2^ ^]^ lasers,^[^ ^3^ ^]^ and data storage.^[^ ^4^ ^]^ The utilization of short‐lifetime singlet and long‐lifetime triplet excitons enables the phosphorescent organic light‐emitting diodes to achieve an internal quantum efficiency (IQE) approaching 100%. Moreover, the long lifetime of the triplet excitons owing to the spin‐forbidden characteristics can increase the exciton diffusion distances and consequently alleviate the strong morphology‐ and thickness‐dependence of the power conversion efficiency (PCE) in OSCs.^[^ ^5^ ^]^ In principle, a viable strategy to obtain triplet excitons is to enhance the intersystem crossing from the S_1_ to the T_1_.^[^ ^6^, ^7^ ^]^ Currently, triplet excitons‐involved organic materials such as organic phosphorescent and thermally activated delayed fluorescence (TADF) materials continue to attract considerable interest,^[^ ^8^, ^9^, ^10^, ^11^ ^]^ but rarely are investigated in OSCs field.

**Figure 1:**
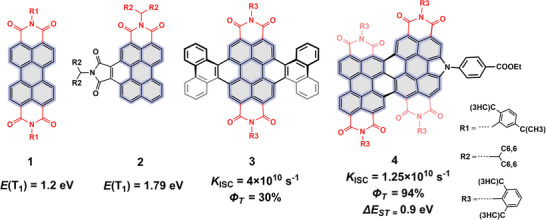
Examples of rylene imides with adjustable *Φ*_ISC,_ *Φ*_T_, and *E*_T1_.

Perylene diimides, composed of two six‐membered carboxylic imide rings, have triggered active research for application in photovoltaic devices, light‐emitting diodes, fluorescent sensors and lasers, due to their flexible reaction sites and interesting photoelectronic properties. As an excellent *n*‐type organic semiconductor materials, their luminescent property and photophysical characteristic, have been extensively investigated in prior studies.^[^ ^12^, ^13^, ^14^ ^]^ For PDI molecule, the unsubstituted monomer 1 shows a high fluorescence quantum yield (*Φ*_F_ = 93%) with a negligible intersystem crossing (ISC) efficiency; whereas, efficient substituent groups attached at the *bay*‐position of PDI skeleton, can perturb the molecular symmetry and thus the inherent structural characteristics. Therefore, efficient ISC, along with adjustable triplet state energy level (*E*_T1_), could be governed by these constituent functionalities, as shown in **Figure** **1**. Of these, the asymmetrically substituted phosphorescent derivative, molecule 2 displayed an elevated *E*_T1_ up to 1.79 eV in DCM‐EtOH glass at 77 K, compared with that of 1.20 eV for PDI monomer 1.^[^ ^15^, ^16^ ^]^ Additionally, a higher intersystem crossing rate (*k*_ISC_) as high as 4 × 10^10^ s^–1^ was further obtained in the doubly twisted fused monomer 3, whose triplet quantum yield (*Φ*_T_) is up to 30%, caused by Herzberg–Teller vibronic coupling.^[^ ^17^ ^]^ Fu et al. observed an ISC efficiency (*Φ*_ISC_) up to 86% via attaching a strong electron donor into the bay‐position of PDI skeleton, with a fast ISC with ≈80 ps;^[^ ^18^ ^]^ furthermore, the fused PDI dimer 4 displayed *Φ*_ISC_ = 94% and triplet state quantum yield (*Φ*_T_) up to 94% and an Δ*E*_ST_ of ≈0.9 eV.^[^ ^19^ ^]^ Therefore, significant triplet excitons, spontaneously generated via fast ISC process from S_1_ state, inherently exist in the state‐of‐the‐art 3D twisted PDI derivatives, upon photoexcitation, but with a *E*_T1_ of ≈1.2 eV.

To date, the great development of synthetic chemistry for rylene core, has provided vast space for progress in PDI molecular evolution, endowing PDI derivatives to be the best wide‐bandgap nonfullerene electron acceptors. Whereas, for OSCs, if the inherent triplet state displays a low‐lying energy than that of charge‐transfer excitons (CTE) at donor–acceptor (D–A) interface, namely *E*_T1_ < *E*_CTE_, these triplet excitons generated via ISC from S_1_ state, will suffer from serious quenching without diffusing into the interfacial CTE and thus leading to serious geminate recombination. This is incompatible with the fact that current 3D fully‐conjugated twisted PDI‐based OSCs possess a high IQE exceeding 90%.^[^ ^20^ ^]^ Furthermore, current relative investigation are mainly focused on the relationships between chemical structure and molecular orbital energy level, charge transfer mobility, as well as the phase separation morphology between electron donors. It is highly demanded, therefore, to establish a network architecture that can distinguish the efficient charge generation process of twisted PDI‐based OSCs with inherent triplet excitons, as well as the link between distinct molecular geometry, excited state electronic distribution, and molecular luminescence mechanism.

**Figure 2:**
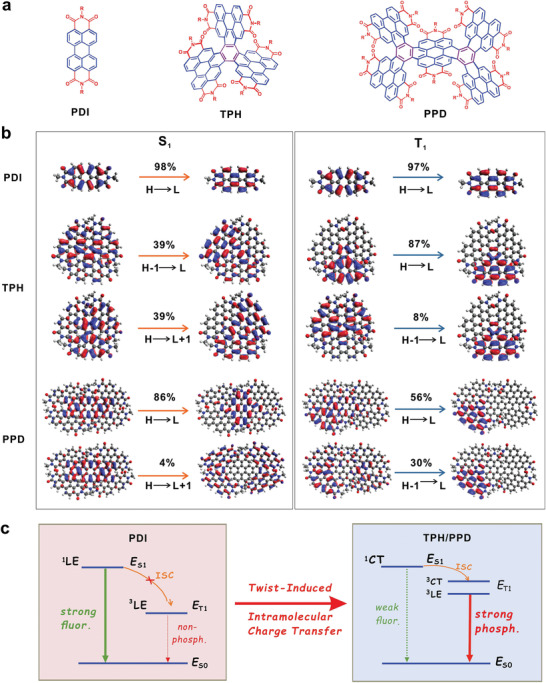
Calculated frontier orbitals transition proportion. a) Chemical structure of PDI, TPH, and PPD. b) Calculated frontier orbitals transition proportion of the S_1_ states and T_1_ states at the PBE0‐optimized S_1_ and T_1_ geometries for PDI, TPH, and PPD molecules. c) The simplified Jablonski diagram describes the key photophysical processes in PDI and TPH/PPD.

In this study, two high‐triplet‐energy rigid propeller‐like PDI electron acceptors with a prominent RTP luminescent behavior were first reported. We systematically investigate the impact of 3D fully‐conjugated twisted rigid molecular geometry on the excited state electron characteristics as well as on the photovoltaic performance, via adopting “three‐bladed propeller” TPH^[^ ^21^ ^]^ and “four‐bladed propeller” PPD^[^ ^22^ ^]^ molecules as electron acceptor and P3TEA as polymer donor. Their chemical structures are as shown in **Figure** **2** and Figure S1 in the Supporting Information. Density functional theory (DFT) calculations reveal that the twisted molecular configurations of TPH and PPD significantly alter the electronic characteristics of the S_1_ and T_1_ states, thereby strengthening their spin–orbit coupling (SOC). As a result, the significant charge transfer feature results in quantitative quenching of the highly fluorescent PDI chromophore, and the localized excitation for T_1_ state facilitates the formation of a long‐lived localized room‐temperature triplet exciton with an energy up to 2.04 eV and lifetime of 66 µs. Above all, even in P3TEA‐based real solid devices, triplet excitons are also detected that derived from the TPH and PPD. The wavefunction delocalization feature for the interfacial^3^CTE at donor–acceptor interface, guarantees their efficient dissociation into free charges, kinetically outcompeting relaxation to the low‐lying triplet exciton formation in narrow‐bandgap P3TEA; and therefore, a high IQE and excellent PCE were observed.

## 2 Results and Discussion

### 2.1 Theoretical Calculation

The geometry optimizations, energy, and frequency calculations were performed for the ground state (S_0_) and excited states (S_1_ and T_1_) employing DFT and time‐dependent DFT (TDDFT), respectively. The SOCs between the singlet states and triplet states were obtained via the TDDFT calculations using the quasi‐degenerate perturbation theory.^[^ ^23^ ^]^ The triethylamine solvent was taken into account using the SMD solvation model.^[^ ^24^ ^]^ The PBE0 functional and Def2‐SVP basis set were used for the entire study. The results based on the M06‐2X functional were also presented for benchmarking. The ORCA 4.2 package^[^ ^25^ ^]^ was used for all DFT and TDDFT calculations. The excited state decay (internal conversion and intersystem crossing) rates were calculated using the TVCF formalisms in the MOMAP package.^[^ ^26^ ^]^ Here, the parent PDI was selected as a representative example for comparison.

The calculated energy diagrams and main transition orbitals of the S_1_ and T_1_ states for PDI, TPH, and PPD molecules are shown in Figure 2, Figure S2 and Tables S1–S3 in the Supporting Information. Only one triplet state T_1_ is observed below the S_1_ state for PDI molecule (Figure S2a, Supporting Information) and both the S_1_ and T_1_ states are characterized as *π*–π* excitation that is distributed over the entire molecule (Figure 2b). As a result, the complete overlap of the highest occupied molecule orbital (HOMO) and lowest unoccupied molecule orbital (LUMO) leads to a large exchange energy, which results in a large energy gap between S_1_ and T_1_, Δ*E*_ST_. Meanwhile, the S_1_ and T_1_ states have similar localized electronic characteristics with a high radiative transition rate (1.45 × 10^8^ s^–1^) from S_1_ to S_0_ and a very low ISC rate (2.68 × 10^–4^ s^–1^) from S_1_ to T_1_, as well as a vanishing SOC constant of 0.0018 cm^–1^, as shown in **Table** **1** and Figure S2a in the Supporting Information. Consequently, the PDI molecule exhibits a high fluorescence quantum yield, a Δ*E*_ST_ as large as 1.2 eV, and extremely weak phosphorescence response with a low *E*_T1_ of ≈1.20 eV. This data is consistent with the experimental results reported in previous studies.^[^ ^16^ ^]^

**Table 1:** Calculated radiative rate *k*_r_ and nonradiative rate *k_n_*_r_ of S_1_→S_0_ or T_1_→S_0_ as well as the intersystem crossing rate *k*_ISC_ of S_1_→T_1_ for PDI, TPH and PPD at 298 and 77 K

	S_1_–S_0_				S_1_–T_1_		T_1_–S_0_			
	*k*_r_ [s^–1^]		*k_n_*_r_ [s^–1^]		*k*_ISC_ [s^–1^]		*k*_r_ [s^–1^]		*k_n_*_r_ [s^–1^]	
	298 K	77 K	298 K	77 K	298 K	77 K	298 K	77 K	298 K	77 K
PDI	1.44 × 10^8^	1.45 × 10^8^	1.07 × 10^3^	1.41 × 10^3^	3.24 × 10^–4^	2.68 × 10^–4^	6.37 × 10^–4^	6.40 × 10^–4^	9.37 × 10^–3^	7.21 × 10^–3^
TPH	1.02 × 10^3^	1.08 × 10^3^	4.54 × 10^5^	4.13 × 10^4^	2.50 × 10^7^	2.08 × 10^7^	1.03 × 10^–1^	1.05 × 10^–1^	2.32	1.94
PPD	4.39 × 10^6^	4.95 × 10^6^	1.00 × 10^8^	2.74 × 10^6^	1.79 × 10^7^	1.60 × 10^7^	1.34 × 10^–1^	1.42 × 10^–1^	0.92	0.72

However, the excited state characteristics of TPH and PPD are significantly more complicated than those of PDI. First, the S_1_ state of TPH is dominated by a significant CT characteristic (Figure 2b), leading to an imperfect oscillator strength (6.6 × 10^–6^) between S_1_ and S_0_, and thus a weak fluorescence efficiency is expected (Table 1). This stabilization of the CT state is relevant to the usual twist‐induced intramolecular charge‐transfer (TICT)^[^ ^27^ ^]^ effect, leading to a small exchange energy and thereby a small Δ*E*_ST_. Additionally, compared to that of the parent PDI, the PPD molecule also exhibits a suppressed radiative rate of S_1_ through the combination of local excitation (LE) and CT electronic characteristics (Figure 2b). Second, the T_1_ states of both TPH and PPD exhibit different electronic characteristics from those of S_1_ and are still most characterized as the LE of one PDI moiety. The different electronic characteristics between S_1_ and T_1_ lead to relatively high SOC coefficients^[^ ^28^ ^]^ (0.31 cm^–1^ for TPH and 0.22 cm^–1^ for PPD), and the corresponding ISC rates are significantly enhanced to 2.50 × 10^7^ s^–1^ for TPH and 1.79 × 10^7^ s^–1^ for PPD (Table 1). Moreover, compared to the PDI molecule, more energetically high‐lying triplet states (T_2_–T_5_ for TPH and T_2_–T_4_ for PPD) are below the S_1_ state, which also present more efficient channels for the ISC from S_1_ to the triplet states (Figure S2, Supporting Information). In addition to the LE character, the T_1_ states of TPH and PPD also show some CT character (Figure 2b), which can explain the increase in T_1_ energy in comparation to that of the PDI molecule, as more energy is required to overcome the Coulomb binding force between the electron and hole during their separation process to form the CT state. This also can be confirmed from the frontier orbital energies as summarized in Table S1 in the Supporting Information. Finally, the calculated radiative rates of T_1_ are comparable to the ISC rates from T_1_ to S_0_ for the TPH and PPD molecules. This affords the TPH and PPD molecules with calculated phosphorescent quantum yields of ≈4% and 2%, respectively, according to the following equations

(1) $$\emptyset_{p}=\emptyset_{\mathrm{isc}}\;k_{rT_{1}-S_{0}}\tau_{p}$$

(2) $$\emptyset_{\mathrm{isc}}=k_{\mathrm{isc}\;S_{1}-T_{1}}/\left(k_{r\;S_{1}-S_{0}}+k_{nr\;S_{1}-S_{0}}+k_{\mathrm{isc}\;S_{1}-T_{1}}\right)$$

(3) $$\tau_{p}=\;1/\left(k_{r\;T_{1}-S_{0}}+k_{nr\;T_{1}-S_{0}}\right)$$

Here, ∅_p_ is the phosphorescence quantum yield, ∅_isc_ is the quantum efficiency of the intersystem crossing from S_1_ to T*_n_* states, and *τ*_p_ is the phosphorescence lifetime. $k_{r\;S_{1}-S_{0}}$ and $k_{nr\;S_{1}-S_{0}}$ are the radiative and nonradiative rates of S_1_→S_0_, respectively. $k_{\mathrm{isc}\;S_{1}-T_{1}}$ and $k_{nr\;T_{1}-S_{0}}$are the ISC rates of S_1_→T_1_ and T_1_→S_0_, respectively. Therefore, this provides strong evidence for the observed red‐phosphorescent emissions in the TPH and PPD solutions.

Based on the above results, it can be concluded that both the TPH and PPD molecules have analogous ground or excited state characteristics. Their minimization of the HOMO–LUMO spatial overlap plus with the suppressed radiative transition rate of S_1_, enhanced ISC rate and strengthened radiative rate of T_1_, would endow them with a bright long‐lived phosphorescent emission behavior.

### 2.2 Photophysical Property

Now we have identified the fast generation mechanism of triplet excitons in TPH and PPD, the key objective of this work is to determine the position of triplet states of these two electron acceptors via phosphorescence measurement directly and unambiguously using a spectrometer equipped with a liquid‐nitrogen‐cooled Dewars, which is of significance for OSCs. Here, we used 1,3‐bis(9‐carbazolyl)benzene (mCP)^[^ ^29^ ^]^ as the phosphorescent host material with a high *E*_T1_ of 2.91 eV and used a mixed solvent (triethylamine^[^ ^30^ ^]^:dichloromethane = 5:1, TEA/DCM) to perform the phosphorescence measurement. Both TPH and PPD present absorption responses from 300 to ≈600 nm in TEA/DCM (Figure S3, Supporting Information), identical to their spectra in chloroform as reported.^[^ ^21^, ^22^ ^]^ When excited using a 350‐nm laser source, mCP is pumped to the excited state, followed by a fast triplet energy transfer to the T_1_ states of TPH and PPD (if possible). As displayed in Figure S4b,d (Supporting Information), the steady‐state luminescence spectra of TPH and PPD display nearly identical shapes to those of the solutions without mCP. The S_1_ state for TPH and PPD were located at 2.27 and 2.20 eV, respectively, from the onset of photoluminescence at 298 K. In addition, emission scans over a wavelength range of 370–1500 nm is performed and both compounds exhibit three main emission bands at 550–750 nm without detectable emission in the near‐infrared region.

**Figure 3:**
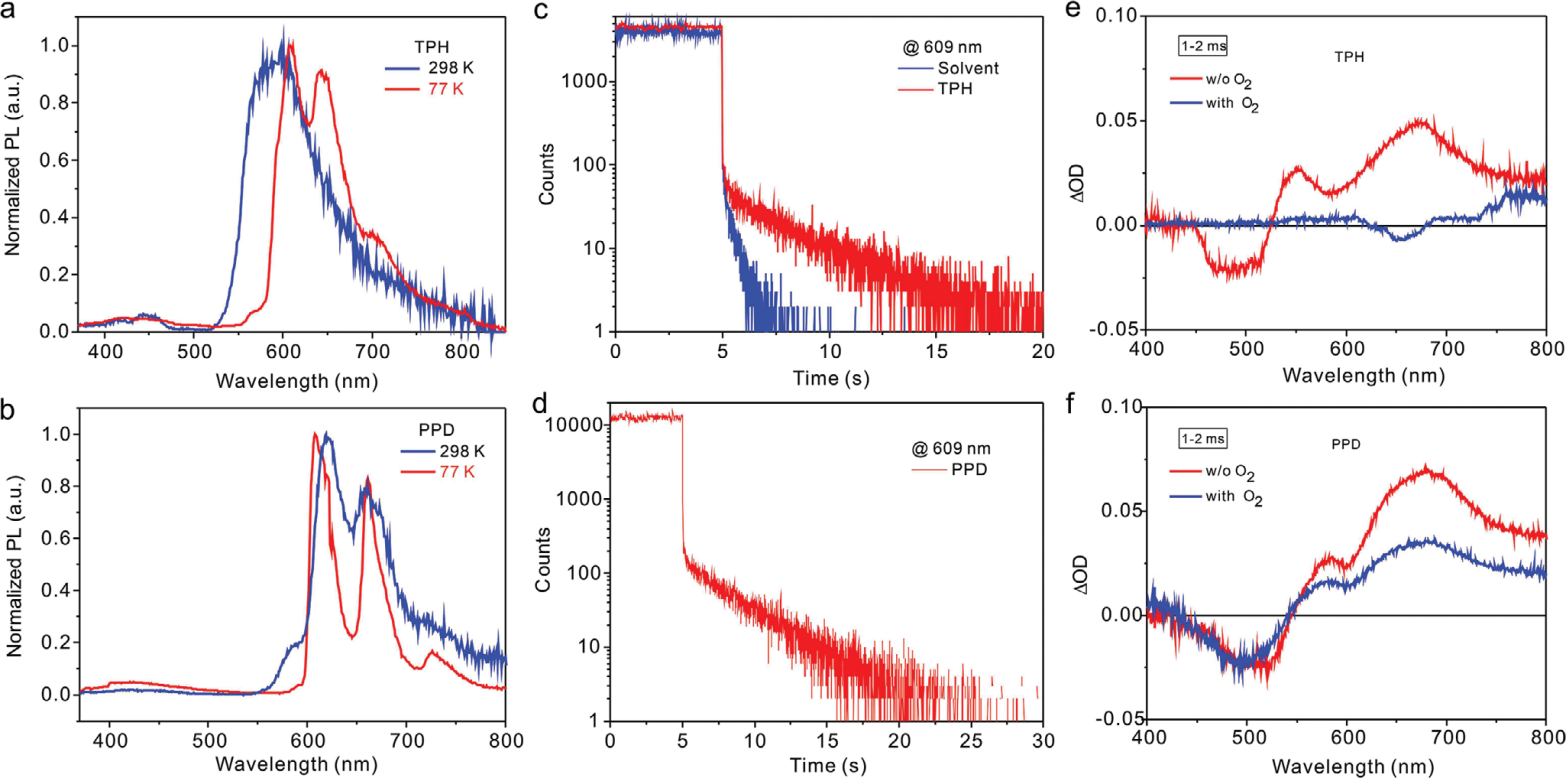
Emission spectra and kinetic curves. a,b) Emission spectra (*λ*_exc_ = 350 nm) of mCP‐sensitized TPH and PPD (mCP:acceptor = 4:1, wt/wt) in TEA/DCM solution (TEA:DCM = 5:1, v/v) at room temperature and at 77 K; c,d) Kinetic curves of mCP‐sensitized TPH and PPD in TEA/DCM solution under the excitation of a microsecond lamp (*λ*_exc_ = 350 nm) at 77K; e,f) Nanosecond transient absorption spectroscopy of TPH and PPD in TEA/DCM with or without oxygen at 298 K.

Furthermore, compared to the luminescence spectrum of TPH at 298 K, its luminescence spectrum at 77 K shows a slight red shift with three peaks at 609, 643, and 702 nm, as displayed in **Figure** **3**a. For the PPD solution, the luminescence spectrum at 298 K almost overlaps with that at 77 K (Figure 3b). Interestingly, no obvious displacement difference is detected in the prompt and delayed emissions, and only a slight variation in intensity is observed, even with 5 ms delay time, as shown in Figure S4a,c in the Supporting Information. This result reveals that these three emission bands mainly correspond to the long‐lived emission at 77 K. From the calculation data, it can be concluded that the phosphorescent spectrum of the TPH or PPD molecule is derived from the lowest triplet excited state, T_1_. The kinetic profile of PPD at 609 nm reveals that the PPD molecule in glassy matrix at 77 K has a lifetime of 3.24 s (Figure 3d). Similarly, the lifetime of the luminescence spectrum for the sensitized TPH molecule at 609 nm is determined as 3.02 s (Figure 3b). This reveals that the orbital energy levels of the singlet excited states and triplet excited states are highly hybridized.

To identify the component characteristics, the PL spectra of PPD solution were measured at different temperatures in the 77–298 K range (Figure S5a, Supporting Information). Herein, with an increase in the temperature from 77 to 298 K, the PL intensities decrease significantly, rather than a response of strengthened PL response with increased temperature as featured in TADF materials. The increase of the overall emission intensity at 77 K is attributed to the suppression of the nonradiative recombination of the excited states and thermal equilibrium of the triplet states. Therefore, the PL emission at 609 nm results from phosphorescence as well as prompt fluorescence, rather than the delayed fluorescence. These experimental results provide clear evidence for the production of the triplet states in the TPH and PPD molecules at 77 K and the T_1_ state for TPH and PPD were located at 2.13 and 2.07 eV, respectively, from the onset of phosphorescence spectra at 77 K. Herein, the singlet–triplet splitting (Δ*E*_ST_) in TPH and PPD is narrowed into 0.14 and 0.13 eV, respectively.

Nanosecond transient absorption spectroscopy was also performed to further reveal if the long‐lived triplet characteristics of the TPH and PPD molecules existed in the solution at room temperature (Figure 3e,f). The transient absorption spectroscopy data of PPD and TPH solution show a negative absorption or bleach feature at range of 400–550 nm, whereas the excited state absorption characteristics are observed at >550 nm. Even when the delay time is extended to 1–2 ms, the positive absorption signals at 550–800 nm are also visible. When oxygen is bubbled into the liquids, the transient absorption intensity of PPD in TEA/DCM decreases significantly, and the long‐life species of TPH is almost completely quenched. The slow emission components are undoubtedly associated with phosphorescence that persisted with a lifetime of 65.7 µs (Figure S5b, Supporting Information). Therefore, these broken spatial symmetries for TPH and PPD results in a highly emissive triplet excitons that decay directly to ground state and generate phosphorescence,^[^ ^31^ ^]^ as shown in Figure 2c.

### 2.3 Photovoltaic Property

**Figure 4:**
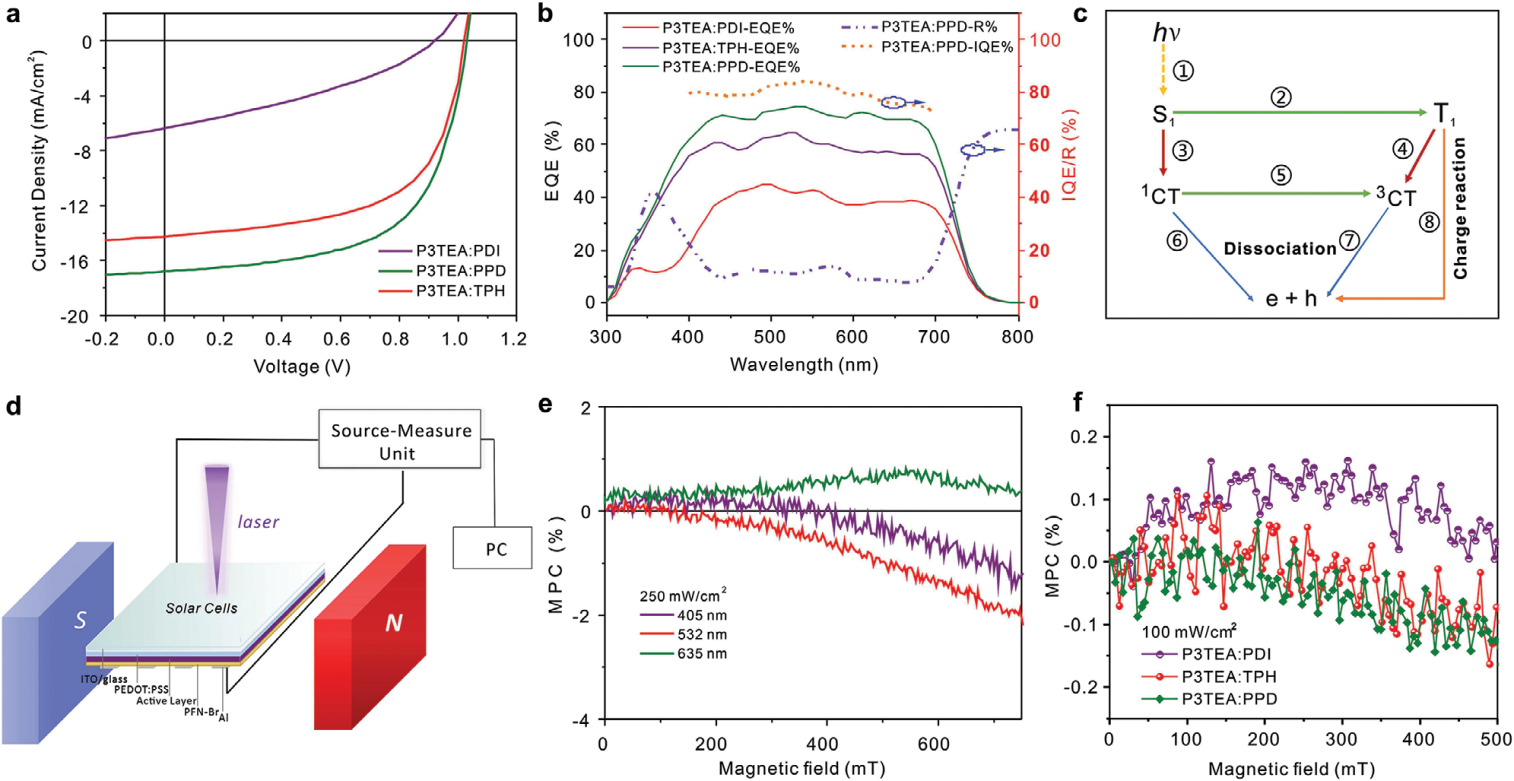
Device performance and spin‐dependent electron–hole dissociation mechanism. a) *J*–*V* characteristics of PDI‐, TPH‐, and PPD‐based solar cells. b) EQE response of PDI‐, TPH‐, and PPD‐based solar cells and IQE, reflection spectrum of PPD‐based cells. c) Two photovoltaic channels: electron–hole dissociation dominated by singlet excitons and exciton–charge reaction dominated by triplet excitons; the detailed channels are as following:①generation of excitons; ②intersystem crossing of excitons; ③diffusion of singlet excitons; ④diffusion of triplet excitons; ⑤intersystem crossing of polaron‐pairs; ⑥dissociation of singlet polaron‐pairs; ⑦dissociation of triplet polaron‐pairs; ⑧triplet exciton–charge reaction. d) Experimental setup for solar cell devices with magnetic and electronic field. Results of magnetic‐field dependence of photocurrent for e) P3TEA:PPD‐based devices under different photoexcited wavelength with 250 mW cm^−2^ illumination intensity and for f) P3TEA:PDI‐, P3TEA:TPH‐, P3TEA:PPD‐based devices under 405‐nm‐laser with 100 mW cm^−2^ illumination intensity.

Considering the critical role of triplet excitons in organic electronics, as well as the excellent photophysical properties of TPH and PPD, their unique photovoltaic applications were investigated. First, solar cells with the conventional ITO/PEDOT:PSS/active layer/PFN‐Br/Al architecture were prepared by employing P3TEA^[^ ^32^ ^]^ with the *E*_S1_ of 1.74 eV, as the donor material. Of these, the LUMO values of PDI, TPH, and PPD are estimated to be −3.88, −3.83, and −3.82 eV, respectively, from the first reduction waves in Figure S6 in the Supporting Information. Their HOMO levels are calculated to be −6.17, −6.02, and −5.82 eV, respectively, according to their optical bandgap.^[^ ^15^, ^20^, ^21^ ^]^ According to the absorption spectra as shown in Figure S7 (Supporting Information), the absorption spectrum of P3TEA is highly complementary with that of TPH and PPD. The corresponding photovoltaic parameters at different experimental conditions are summarized in Table S4 in the Supporting Information. Interestingly, the P3TEA:TPH‐ and P3TEA:PPD‐based solar cells exhibit excellent PCEs of 8.76% and 10.55%, respectively, with an outstanding open‐circuit voltage (*V*_OC_) of 1.03 V. The current density–voltage (*J*–*V*) characteristics, external quantum efficiency (EQE) and IQE curves of the optimal devices are shown in **Figure** **4**a,b. It is clearly demonstrated that the PPD‐based OSCs exhibit an outstanding IQE at range from 300 to 700 nm, with a maximum value of 84% at ≈550 nm.

In order to investigate if the triplet excited state participated in photovoltaic devices, magnetic photocurrent measurement^[^ ^33^, ^34^ ^]^ were carried out, as shown in Figure 4. The generation of charge carries from photoexcited states is sensitive to the external magnetic field, and consequently accounts for the magnetic‐field effects (MFEs) in organic semiconductors. As shown in Figure 4c, there are two photovoltaic channels for excited states to generate photocurrent: singlet exciton‐dominated dissociation (①→③→⑥ or ①→③→⑤→⑦) and triplet exciton‐dominated charge reaction (①→②→④→⑦ or ①→②→⑧) in organic semiconductors.^[^ ^34^ ^]^ For singlet exciton‐dominated dissociation process, an external magnetic field can facilitate the field‐dependent RISC rate from triplet‐charge‐transfer (^3^CT) state to singlet‐charge‐transfer (^1^CT) state and therefore the strengthened singlet‐exciton dissociation leading to a positive magnetic photocurrent change (MPC). Whereas, due to the long‐lived characteristic and high binding energy of triplet excitons (TE), they have sufficient physical contact with trapped charge carriers than singlet‐excitons (SE) and^1^CT/^3^CT. Hence, the external magnetic field could pertub triplet exciton–charge reaction rate constant, if there are triplet excitons generated from singlet excitons through field‐independent ISC. This process will suppress the triplet exciton–charge reaction and thus the release process of trapped charge carriers is suppressed, yielding a negative MPC.^[^ ^35^ ^]^

Herein, various laser masters with different wavelength (405, 532, and 635 nm) and illumination of 100 and 250 mW cm^−2^ were utilized to completely explain the MFE. It was notable that, as shown in Figure 4f, the cells based on TPH or PPD exhibited negative MPC effects, when utilizing a Laser of 405 nm wavelength with 100 mW cm^−2^ illumination intensity to photoexcite the electron acceptors. Whereas, a significant positive MPC signal was observed for the P3TEA:PDI‐based device when performed at the same measurement condition. Generally, the *E*_T1_ of polymer is just located at 0.6–0.7 eV below *E*_S1_,^[^ ^36^ ^]^ namely *E*_T1_ of 1.14 eV in P3TEA. Therefore, we are wondering that if these triplet excitons come from TPH and PPD via ISC process, or stem from the process of free charge recombination (CR) to the P3TEA low‐lying T_1_ state (CR→T_1_). But the fact is that even for the P3TEA:PDI‐based OSCs with prior electron mobility that intend to cause the bimolecular recombination, the MFE signal does not display any presence of triplet excitons. More importantly, when P3TEA was selectively excited by 635 nm laser, the P3TEA:PPD‐based devices exhibited nearly constant positive MPC signal; whereas, persistent negative MPC signal was displayed when excited by 405 and 532 nm lasers (Figure 4e). These results clearly demonstrate the negative MPC effects in TPH‐ or PPD‐based solar cells are exactly involved with the triplet excitons generated via S_1_→T_1_ in TPH and PPD molecules, rather than caused by the process of free charge recombination into T_1_ of P3TEA.

### 2.4 Charge Generation Process

**Figure 5:**
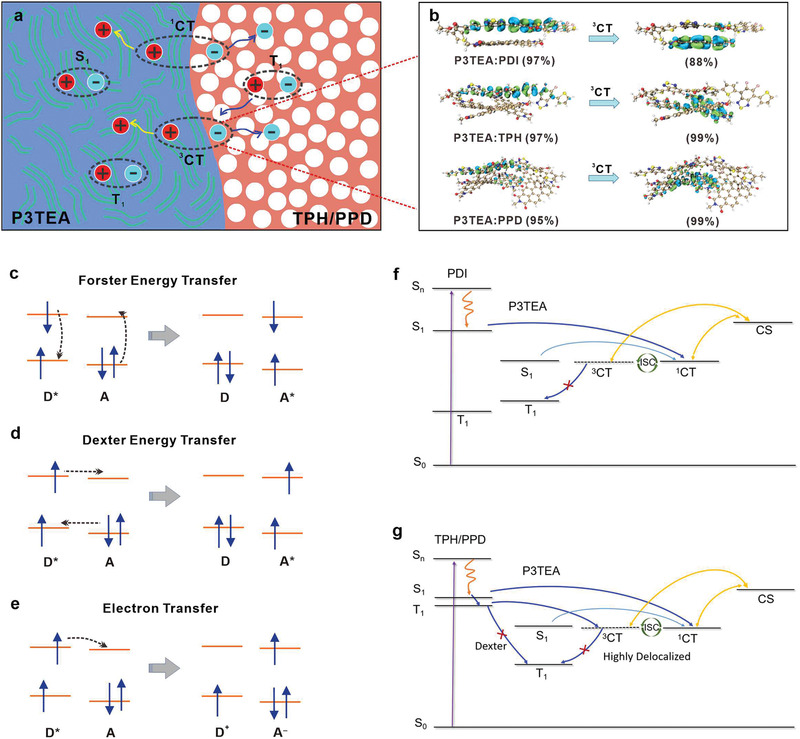
Photophysical process in an OSCs. a) Exciton diffusion and separation process; b) Charge distribution in the^3^CT state configuration; c) Förster energy transfer between singlet excitons; d) Dexter energy transfer between triplet excitons; e) Electron transfer from an excited donor (D*) to a neutral acceptor; f) Jablonski state diagrams of electronic transitions for PDI‐based solar cells; g) Jablonski state diagrams of electronic transitions for TPH‐, PPD‐based solar cells. The energies of singlet (S_0_ and S_1_) and triplet (T_1_) states are scaled vertically. Absorption (purple line), internal conversion (orange line), intersystem crossing (blue line), and charge recombination (yellow line).

Notably, from the perspective of energy level, such fast formation of photogenerated TEs of TPH/PPD may experience two possible pathway: ①transferring into P3TEA TE via a Dexter energy transfer (T_1_→T_1_, **Figure** **5**d); ②subsequently diffusing into the ^3^CTE at D‐A interface via an electron transfer (T_1_→^3^CT) (Figure 5a). The first channel is the terminal energy loss for this system, competing the subsequent formation of ^3^CTE as well as of free carriers. But from a practical point of view, this Dexter energy transfer is only occurs at the significant overlap of molecular orbitals, meaning the distance between donor and acceptor are within 1 nm, merely at D‐A interfaces.^[^ ^5^, ^37^, ^38^ ^]^ Furthermore, four‐orbital related energy transfer feature demonstrates the lower energy transfer rate than that of two‐orbital related ultrafast electron transfer process for the ^3^CTE formation (Figure 5e). That is, although the large spectral overlap between the emission spectrum of PDI‐based electron acceptor and the absorption spectrum of narrow‐bandgap donor, the electron transfer between TPH/PPD and P3TEA also outcompetes to energy transfer process, which is also proved by Singh et al. in 2015.^[^ ^39^ ^]^ Meanwhile, highly sensitive Fourier‐transform photocurrent spectroscopy (FTPS) and electroluminescence (EL) measurements^[^ ^40^, ^41^, ^42^ ^]^ revealed the ^3^CT/^1^CT states at the P3TEA:TPH and P3TEA:PPD interfaces are located at 1.74 eV and that is 1.69 eV for P3TEA/PDI interface(Figure S8, Supporting Information). The faster T_1_→^3^CT intersystem crossing rate, plus with *E*_T1_>*E*_CTE_, guarantee the efficient TEs diffusion into ^3^CTE.

Herein, quantum‐chemical excited state calculations of charge‐transfer excitons for P3TEA:PDI, P3TEA:TPH, and P3TEA:PPD were performed to infer the nature of their ^3^CTE. As shown in Figure 5b, the wavefunction of the lowest ^3^CT states in these three blend films, are delocalized nearly completely between P3TEA and acceptors. Of these, the number on the left and right sides are the percentage of transition orbital contributed from the donor, and from the acceptor, respectively. This demonstrated that the well‐ordered acceptor molecules with encouraging wavefunction delocalization, benefit for a faster ^3^CTE dissociation into free charges, than relaxation to low‐lying T_1_ state.^[^ ^43^, ^44^ ^]^ The more delocalized ^3^CTE in P3TEA:TPH and P3TEA:PPD blend film than that of in P3TEA:PDI film, revealed a more efficient charge separation process occurs for P3TEA:TPH and P3TEA:PPD complexes. These delocalized charge wavefunctions of ^3^CTE, plus with low reorganization energies induced by the structural rigidity and suppressed torsion relaxation, facilitate the efficient charge separation of ^3^CTE. This is also proved in recent reported P3TEA:SF‐PDI_2_ system.^[^ ^45^ ^]^ Therefore, even in the case that the *E*_T1_ of P3TEA is located below *E*_CTE_ at D–A interface, a relaxation from ^3^CT state to the P3TEA T_1_ state is also avoidable through intentional molecular modification.

Based on the above data, the resulting Jablonski state diagram describing the charge generation process in the P3TEA:rylene‐based devices are proposed in Figure 5f,g. The higher distribution of *E*_T1_ in TPH or PPD molecules relative to the interfacial ^3^CT/^1^CT states results in an efficient triplet exciton dissociation, rather than affording a major quenching pathway. Consequently, OSCs based on P3TEA:TPH and P3TEA:PPD, exhibit higher charge collection probabilities, *P*(E,T) of 94.3% and 92.5%, respectively, as shown in Figure S9 in the Supporting Information.

## 3 Conclusion

In conclusion, two room‐temperature phosphorescent PDI‐based electron acceptors with significantly elevated triplet state to ≈2.10 eV, from 1.2 eV for parent PDI monomer, was first reported. In this work, we mainly focus on the key photophysical and photovoltaic processes related on the triplet excitons. For example, we studied excited state electronic characteristics, intersystem crossing, radiative and nonradiative decays, luminescent behavior, and photo‐to‐current pathways. DFT and TDDFT calculations reveal that this asymmetric intramolecular charge transfer effect accelerates the spatial separation of the hole and electron for TPH and PPD molecules. The dominant CT characteristics for S_1_ state and prominent LE features for T_1_ state, lead to a strong SOC. Furthermore, the partial CT characteristics of T_1_ state further minimize the Δ*E*_ST_ as low as ≈0.13 eV and elevate *E*_T1_ to ≈2.10 eV, much higher than 1.74 eV for interfacial ^3^CT state. Specific negative MFEs confirm the presence of TEs in real devices, which is coming from SEs via ISC process in TPH and PPD. These highly‐delocalized characteristics for ^3^CTEs, restrain their rebound into the low‐lying P3TEA T_1_ states. As a result, the wide‐bandgap OSCs based on heavy‐atom‐free RTP materials display an outstanding PCE of 10.55% and excellent charge collection probability of 94%.

To date, the state‐of‐the‐art high‐performing organic photovoltaic materials are usually highly‐twisted and molecular asymmetrical, and thus, it is of great significance to elevate their *E*_T1_ above the *E*_CTE_ at D–A interface. Therefore, a fundamental analysis on the link between distinct molecular architecture, excited state electronic feature, photophysical behavior and thus triplet‐exciton involved photo‐to‐current generation pathway should be considered critically.

## Conflict of Interest

The authors declare no conflict of interest.

## Supporting information

Supporting Information

## Data Availability

The data that support the findings of this study are available from the corresponding author upon reasonable request.
