# Supplementary material for: Heavy‐Atom‐Free Room‐Temperature Phosphorescent Rylene Imide for High‐Performing Organic Photovoltaics
Source: Adv Sci (Weinh). 2021 Nov 23;9(3):2103975. doi: 10.1002/advs.202103975 (PMC8787389; doi:10.1002/advs.202103975)
Supplement: Supplementary file 1 — Supporting Information [file ADVS-9-2103975-s001.pdf]

ADVANCED SCIENCE
Open Access

Supporting Information

for *Adv. Sci.,* DOI: 10.1002/advs. 202103975

Heavy-Atom-Free Room-Temperature Phosphorescent Rylene Imide for High-Performing Organic Photovoltaics

Ningning Liang, Guogang Liu, Deping Hu,* Kai Wang, Yan Li, Tianrui Zhai, Xinping Zhang, Zhigang Shuai, He Yan, Jianhui Hou, Zhaohui Wang**

Supporting Information

Heavy-Atom-Free Room-Temperature Phosphorescent Rylene Imide for High-Performing Organic Photovoltaics

Ningning Liang, Guogang Liu, Deping Hu,* Kai Wang, Yan Li, Tianrui Zhai, Xinping Zhang, Zhigang Shuai, He Yan, Jianhui Hou, Zhaohui Wang**

Table of Contents

1. **Materials**......S2

2. **Photophysical properties**......S2

2.1 **Theoretical calculation**......S2

2.2 **Fluorescence and phosphorescence spectra measurement**......S4

2.3 **Nanosecond transient absorption spectroscopy measurement**......S5

3. **Device Fabrication and Characterization**......S6

3.1 **OPV device fabrication**......S6

3.2 **OPV device characterization**......S7

3.3 **Magnetic-field photocurrent measurement**......S8

3.4 **Energy measurement of CT state**......S8

References......S9

1. Materials

P3TEA, PDI, TPH and PPD were synthesized according to the relevant literature. The chemical structure of P3TEA is as shown in Figure S1. 1,2,4-Trimethylbenzene (1,2,4-TMB, CAS: 95-63-6, 98%) was obtained from TCI Co. The 1,8-Diiodooctane (DIO, 98%) was obtained from Sigma-Aldrich. 9,9-(1,3-phenylene) bis-9H-carbazole (mCP, CAS: 550378-78-4, 98%) was purchased from TCI Co. without further purification.

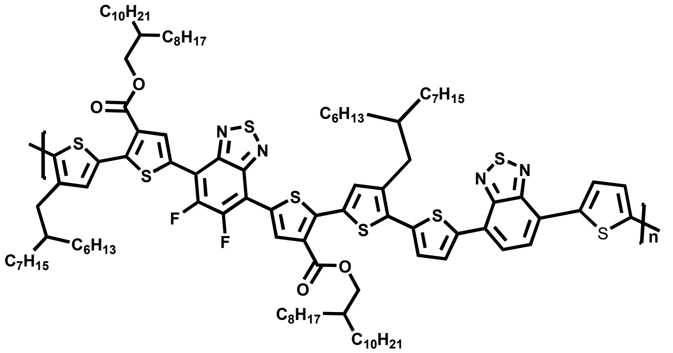

Figure S1. Chemical structure of polymer donor P3TEA.

1. Photophysical properties

2.1 Theoretical calculation

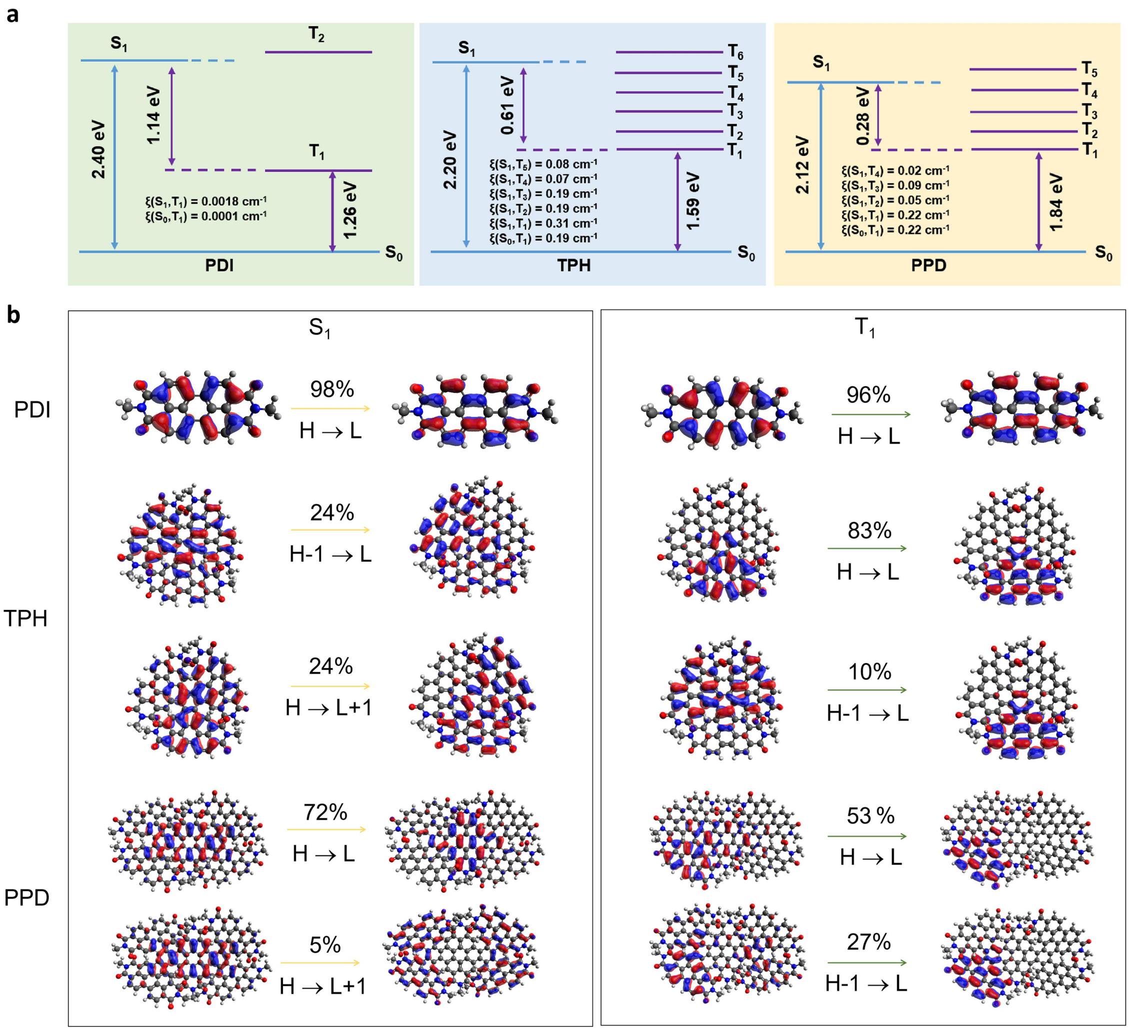

Figure S2. (a) Calculated adiabatic energy diagram and SOC coefficients PDI, TPH and PPD; (b) Calculated frontier orbitals and transition proportion of the S_1 and T_1 states at the M06-2X-optimized S_1 and T_1 geometries for PDI, TPH and PPD molecules.

Table S1. Frontier orbital energies for PDI, TPH and PPD at the optimized S_1 and T_1 geometries at the PBE0 level of theory

Molecule	Geom.	HOMO-1 (eV)	HOMO (eV)	LUMO (eV)	LUMO+1 (eV)	LUMO-HOMO (eV)
PDI	S_1-min	-7.7439	-6.1224	-3.6740	-1.8096	2.4484
	T_1-min	-7.7417	-6.0568	-3.7248	-1.7560	2.332
TPH	S_1-min	-6.2668	-6.2648	-3.4915	-3.4903	2.7733
	T_1-min	-6.3310	-6.1930	-3.6282	-3.4359	2.5648
PPD	S_1-min	-6.3677	-6.1431	-3.5172	-3.4666	2.6259
	T_1-min	-6.2898	-6.1586	-3.6649	-3.4749	2.4937

Table S2. The energy level and the oscillator strength of S_1 excited states for PDI, TPH and PPD at the PBE0 and M06-2X level of theory

Molecule	Functional	S_1-Ead (eV)	S_1-Ead (nm)	S_1-Evt (eV)	S_1-Evt (nm)	S_1-OS
PDI	PBE0	2.40	517	2.24	553	0.67
	M06-2X	2.61	474	2.41	515	0.76
TPH	PBE0	2.20	563	2.14	579	6.6×10^{-6}
	M06-2X	2.64	470	2.51	494	8.4×10^{-6}
PPD	PBE0	2.12	585	2.03	611	3.5×10^{-2}
	M06-2X	2.51	494	2.38	521	3.5×10^{-2}

Table S3. The energy level of T_1 excited states for PDI, TPH and PPD at the PBE0 and M06-2X level of theory

Molecule	Functional	T_1-E_{ad} (eV)	T_1-E_{ad} (nm)	T_1-E_{vt} (eV)	T_1-E_{vt} (nm)
PDI	PBE0	1.26	988	1.01	1228
	M06-2X	1.47	845	1.15	1082
TPH	PBE0	1.59	781	1.36	912
	M06-2X	1.83	677	1.55	801
PPD	PBE0	1.84	674	1.36	912
	M06-2X	2.07	599	1.55	800

2.2 Fluorescence and phosphorescence spectra and lifetimes measurements

The fluorescence and phosphorescence spectra and lifetimes were measured using an Edindurgh FLS980 fluorescence spectrophotometer equipped with a xenon laser arc lamp (Xe900), a microsecond flash lamp (μF900), and a picosecond pulsed diode laser (EPL-375) and a closed-cycle cryostat (CS202*I-DMX-1SS, Advanced Research Systems). The picosecond pulsed diode laser (EPL-375) was used as the excitation source for the fluorescence lifetimes. For phosphorescence lifetimes, the excitation wavelength is 350 nm with the microsecond flash lamp and the solution (10^{-5} mol/L) is bubbled with high purity N_2 for ~20 min to completely rule out oxygen.

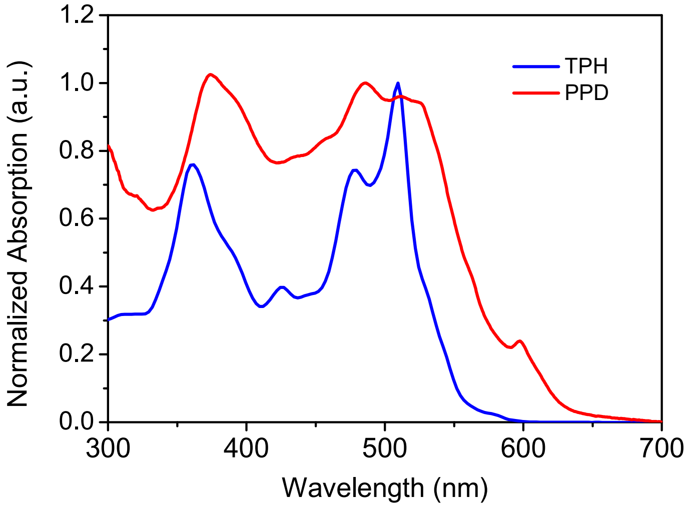

Figure S3. The normalized steady-state absorption spectra of TPH and PPD in dilute TEA/DCM solution (10^{-5} mol/L, TEA:DCM=5:1, v/v) at room temperature.

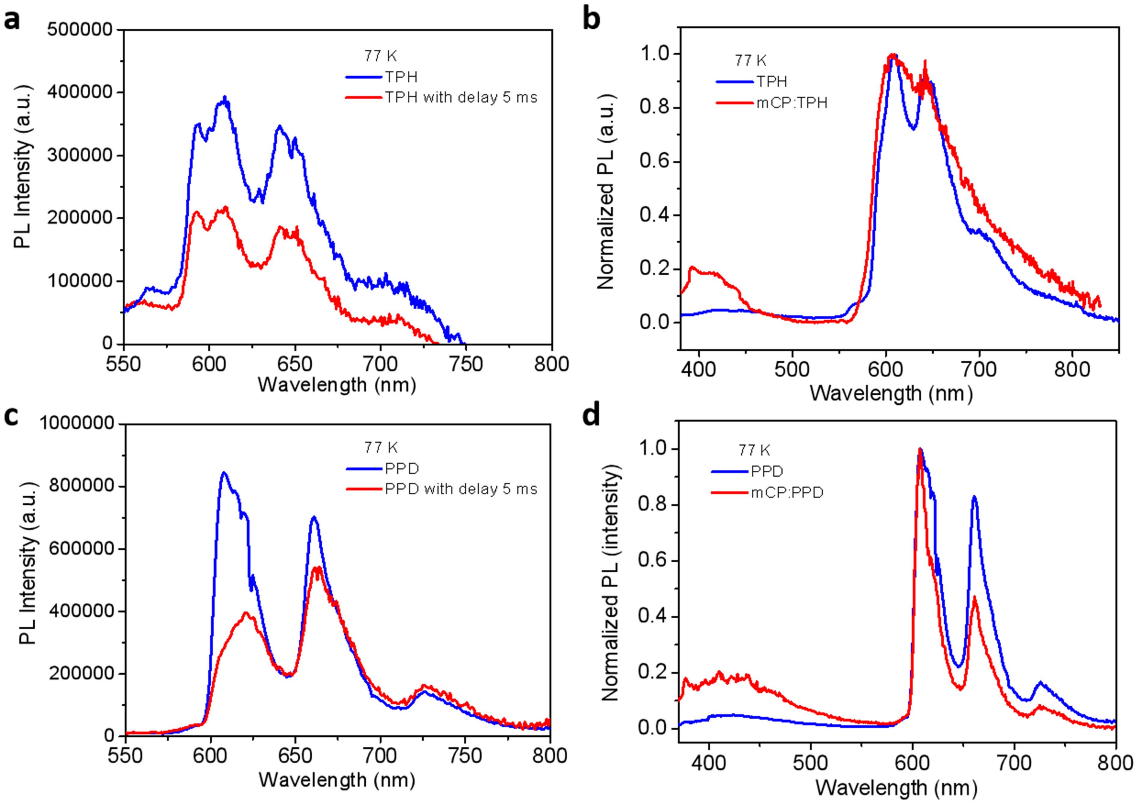

Figure S4. (a)(c) The steady-state and time-resolved (delayed) photoluminescence spectra of TPH and PPD in TEA/DCM solution at 77 K with mCP (TEA:DCM=5:1,v/v, mCP:acceptor=4:1, wt/wt); (b)(d) The steady-state photoluminescence spectra of TPH and PPD in TEA/DCM with and without mCP.

2.3 Nanosecond transient absorption spectroscopy measurement

A pulsed xenon arc lamp was used to provide the analyzing light. The configuration of the monitoring light with respect to the excitation laser pulse is a perpendicular geometry. The liquid samples (1.0 cm quartz cell) were settled on the platform at the intersection of the monitoring light and the excitation pulse. The signals were detected by the Edinburgh LP920 and recorded on the Tektronix TDS 3012B oscilloscope and computer. The triplet lifetimes were obtained by kinetic analysis of the transient absorption with the completely removement of oxygen through bubbling with high purity N_2 for ~20 min. All the spectra were measured at room temperature without further notification.

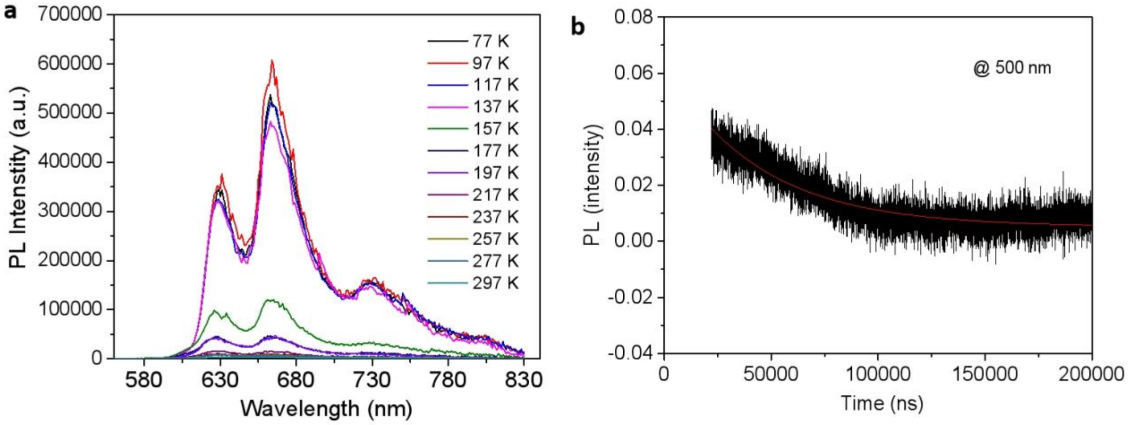

Figure S5. (a) The PL spectra of PPD solution measured at different temperature from 77 K to 298 K; (b) Time-resolved PL-dacay curves for PPD phosphorescence at 298 K.

2. Devices Fabrication and Characterization

3.1 OPV device fabrication

Preparation of anode buffer layer: The ITO-coated glass (15 Ω/□) was cleaned with deionized water, acetone, and isopropanol, respectively, in an ultrasonic bath; and then was treated with UV/ozone cleaning for 20 min. The precursor solution of PEDOT:PSS was stirred at room temperature for about 30 min, and then was spin-coated onto the ITO substrate to obtain a buffer layer with a thickness of 15 nm. Following, the substrates were dried by baking in an oven at 150 °C for 15 min in air.

Preparation of photoactive layers: The blend of P3TEA and different acceptors (PDI, TPH and PPD) was solved into 1,2,4-TMB with 9 mg/mL of polymers and stirred at 90 °C for about 2 hours. The active layer solutions with different D:A ratio and different volume of DIO was then spin-coated onto the top of the PEDOT:PSS layer for ~85-nm-thick active layer film. It was worthwhile to note that the substrates needed to be pre-thermal-treated on the heating-

stage with a temperature of 92 °C for 20 seconds. After that, the active layer films were thermal annealing for 10 minutes at 90 °C.

Preparation of cathode buffer layer: Following, the methanol solution of PFN-Br (0.5 mg/mL) was spin-coated onto the active layer with 3000 rpm. Finally, 100-nm-thick Al layer was evaporated on top of the cathode buffer layer under vacuum at a pressure of 3×10^{-4} Pa. The effective area, namely the overlapping area between the cathode and anode is determined to be ~ 0.04 cm^2.

3.2 OPV device characterization

The *J*–*V* characteristic was performed via the solar simulator (SS-F5-3A, Enlitech) with AM 1.5G spectra, in which the light-intensity was calibrated by the standard silicon solar cell (SRC-2020, Enlitech) at 100 mW/cm^2. The EQE profiles were obtained from a solar cell spectral response measurement system (QE-R3011, Enli Technology Co. Ltd). In addition, the film thickness was measured via a surface profilometer (Dektak XT, Bruker).

Table S4. Photovoltaic Parameters of the solar cells based on different active layers at different condition under AM 1.5G illumination of 100 mW/cm^2

Active layer	D/A Ratio	Additive (v/v, %)	V_{OC} (V)	$J_{SC}{}^{a}$ (mA/cm^2)	$J_{SC}{}^{b}$ (mA/cm^2)	FF	PCE (%)
P3TEA:PPD	1.2:1	W/O	1.02	16.25	--	0.57	9.45
	1:1	W/O	1.02	16.91	--	0.57	9.81
		0.5% DIO	1.03	16.77	15.32	0.61	10.55
	1:1.2	W/O	1.02	14.55	--	0.61	8.94
P3TEA:TPH	1:1	0.5% DIO	1.03	13.95	12.82	0.61	8.76
P3TEA:PDI	1:1	0.5% DIO	0.93	6.54	8.47	0.35	2.10

[a] Obtained from the *J*-*V* curves; [b] integrated from the EQE plots

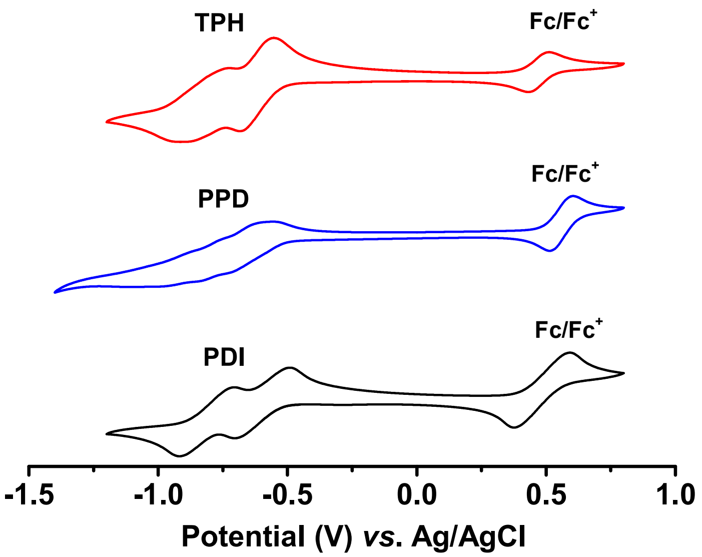

Figure S6. Cyclic voltammetries of PDI, TPH and PPD in CH_2Cl_2 solution.

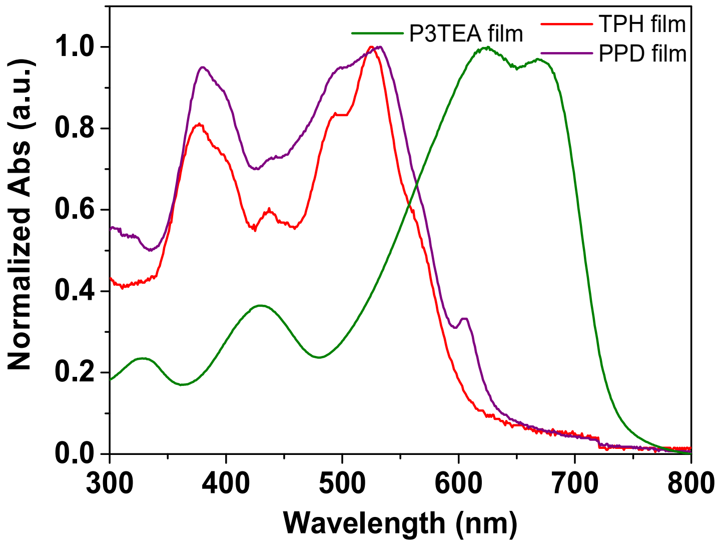

Figure S7. Absorption spectra of TH, PPD and P3TEA film.

3.3 Magnetic-field effect measurement

The class IIIb laser product (Changchun New Industries Optoelectronics Tech CO., Ltd) with a laser of 405 nm and a light density of 250 mA/cm^2. The laser of 405 nm was utilized in order to selectively excite the wide-bandgap PDI acceptors. Here, the experimental setup for the devices with magnetic and electronic field is identical to the literature[1].

3.4 The energy of CT state measurement

FTPS-EQE was performed using a Vertex 70 from Bruker optics, equipped with a QTH lamp, quartz beam splitter and external detector option. SR570, a low noise current amplifier, is used to amplify the photocurrent of the solar cell devices with light modulated by FTIR. In order to be able to use the FTIR's software to collect the photocurrent spectrum, the output voltage of the current amplifier is fed back into the external detector port of the FTIR.

EL spectra were measured through the device (ELCT-3010, Enlitech) by applying external voltage or current sources. In this measurement, the voltage was carried out from 1 to 5 V. The E_{CT} can be determined by fitting the tail of EQE or EL spectra of the CT states according to the following equations[2-4]:

$$\frac{EQE(E)}{E} \approx \exp(-\frac{(E - E_{CT} - \lambda)^2}{4\lambda\kappa T}) \qquad (1)$$

$$\frac{EL(E)}{E^3} \approx \exp(-\frac{(E - E_{CT} + \lambda)^2}{4\lambda\kappa T}) \qquad (2)$$

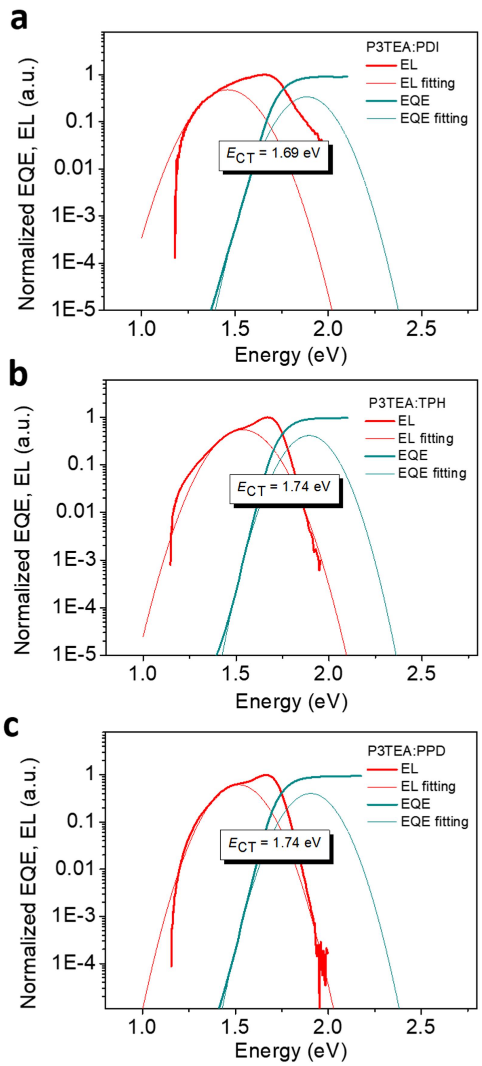

Figure S8. Determination of the E_{CT} values for (a) P3TEA:PDI, (b) P3TEA:TPH and (c) P3TEA:PPD-based devices from the FTPS-EQE (thick green lines) and EL (thick red lines) spectra.

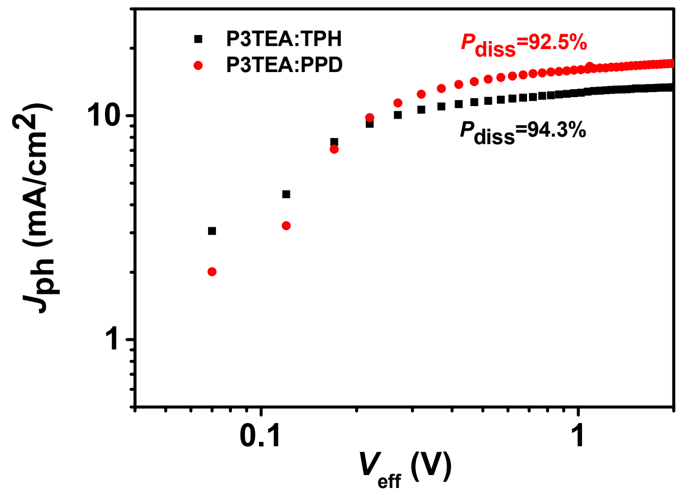

Figure S9. Photocurrent density (J_{ph}) versus effective bias (V_{eff}) curves.

References

(1) Liang, N. N.; Zhu, X. X.; Zheng, Z.; Meng, D.; Liu, G. G.; Zhang, J. Q.; Li, S. S.; Li, Y.; Hou, J. H.; Hu, B.; Wang, Z. H. *Chem. Mater.* **2019**, *31*, 3636.

(2) Vandewal, K.; Benduhn, J.; Nikolis, V. C. *Sustainable Energy Fuels* **2018**, *2*, 538−544.

(3) Marcus, R. A. *J. Phys. Chem.* **1989**, *93*, 3078−3086.

(4) Gould, I. R.; Noukakis, D.; Gomez-Jahn, L.; Young, R. H.; Goodman, J. L.; Farid, S. *Chem. Phys.* **1993**, *176*, 439−456.
